# MetaTOR: A Computational Pipeline to Recover High-Quality Metagenomic Bins From Mammalian Gut Proximity-Ligation (meta3C) Libraries

**DOI:** 10.3389/fgene.2019.00753

**Published:** 2019-08-20

**Authors:** Lyam Baudry, Théo Foutel-Rodier, Agnès Thierry, Romain Koszul, Martial Marbouty

**Affiliations:** ^1^Institut Pasteur, Unité Régulation Spatiale des Génomes, UMR3525, CNRS, Paris, France; ^2^Institut Pasteur, Center of Bioinformatics, Biostatistics and Integrative Biology (C3BI), Paris, France; ^3^Sorbonne Université, Collège Doctoral, Paris, France

**Keywords:** metagenomics Hi-C, gut microbiome, Hi-C, metagenomics binning, metagenomic analysis, binning algorithm, metagenome-assembled genomes

## Abstract

Characterizing the complete genomic structure of complex microbial communities would represent a key step toward the understanding of their diversity, dynamics, and evolution. Current metagenomics approaches aiming at this goal are typically done by analyzing millions of short DNA sequences directly extracted from the environment. New experimental and computational approaches are constantly sought for to improve the analysis and interpretation of such data. We developed MetaTOR, an open-source computational solution that bins DNA contigs into individual genomes according to their 3D contact frequencies. Those contacts are quantified by chromosome conformation capture experiments (3C, Hi-C), also known as proximity-ligation approaches, applied to metagenomics samples (meta3C). MetaTOR was applied on 20 meta3C libraries of mice gut microbiota. We quantified the program ability to recover high-quality metagenome-assembled genomes (MAGs) from metagenomic assemblies generated directly from the meta3C libraries. Whereas nine high-quality MAGs are identified in the 148-Mb assembly generated using a single meta3C library, MetaTOR identifies 82 high-quality MAGs in the 763-Mb assembly generated from the merged 20 meta3C libraries, corresponding to nearly a third of the total assembly. Compared to the hybrid binning softwares MetaBAT or CONCOCT, MetaTOR recovered three times more high-quality MAGs. These results underline the potential of 3C-/Hi-C-based approaches in metagenomic projects.

## Introduction

Microbial communities hold important roles in ecosystems regulation (Philippot et al., 2013; Edbeib et al., 2016; Coutinho et al., 2018; Rosado et al., 2019), such as the human gut (Cho and Blaser, 2012). Understanding the behaviors of these communities is a complex task, and one important step toward this objective relies on the characterization of the genomes of the different species within (Long et al., 2016). Indeed, the genome sequence allows to infer metabolic pathways and, by extension, provide indications about the species lifestyle in the environment. Supported by high-throughput sequencing technologies dropping costs and backed by increasingly powerful computational resources, the field of metagenomics aims at exploring ecosystems through the analysis of DNA sequences extracted directly from the environment to gain insights on microbial population diversity and dynamics (Spang et al., 2015; Hug et al., 2016; Paez-Espino et al., 2016; Castelle and Banfield, 2018). Characterizing complete or near-complete genomes remains however difficult to achieve, depending to some extent to the popularity and complexity of the ecosystem studied (Olson et al., 2017; Quince et al., 2017; Sieber et al., 2018). An important aspect of metagenomics studies therefore consists in developing computation approaches to characterize genomes in metagenomics data (Albertsen et al., 2013; Alneberg et al., 2014; Frank et al., 2016; Sieber et al., 2018).

Most computational approaches rely on the composition and/or co-abundance of sequences recovered from multiple samples to pool (bin) them together (Alneberg et al., 2014; Wu et al., 2014; Kang et al., 2015; Lu et al., 2017; Graham et al., 2017; Laczny et al., 2017). Composition-based method groups together sequences that display similar metrics, such as GC content and/or tetra- and/or penta-nucleotide frequencies. Co-abundance-based approaches trace the relative amount of sequences over multiple samples and group together those with similar coverage variation. Co-abundance is very effective when multiple samples of the same ecosystem are available under different conditions. Today, most metagenomics binning pipeline consists in hybrid approaches combining both strategies to improve the confidence of the resulting sequences bins (Alneberg et al., 2014; Wu et al., 2014; Kang et al., 2015; Graham et al., 2017; Lu et al., 2017). However, caveats and limitations remain. First, grouping sequences based on their similarities imply a strong assumption regarding the homogeneity of the genomes’ composition. This hypothesis is therefore not valid when horizontal transfer or introgression of genetic material takes place between species with (highly) divergent sequence compositions. For instance, the GC content of prophages and of their bacterial genomes host can differ widely. Co-abundance-based methods require multiple samples and large amounts of data to be fully effective, which can be impractical and/or costly. In addition, if several multiple species share the same genetic elements, co-abundance-based methods will also fail to identify the association of these elements with the different species.

Novel technologies, such as single-cell (Ji et al., 2017), long reads (Frank et al., 2016) or proximity ligation/chromosome conformation capture (3C) (reviewed in Marbouty and Koszul, 2015; Flot et al., 2015), hold the potential to address some of these limitations. The latter approach, dubbed meta3C from the original 3C approach (Dekker et al., 2002), aims at quantifying and exploiting collisions between DNA loci over a population of species to identify those that share the same cellular compartment. Sequences belonging to the same genome display enriched contact frequencies compared to those belonging to different genomes, as shown by applying meta3C on controlled mixes of species (Burton et al., 2014; Beitel et al., 2014; Marbouty et al., 2014). Besides controlled mixes, meta3C successfully reconstructed genomes from truly unknown and complex ecosystems as well (Marbouty et al., 2014; Marbouty et al., 2017; Stewart et al., 2018). Not only near-complete genomes from microorganisms can be recovered from a single experiment, but additional information about the genomic structure of these microbial populations can be recovered as well, including plasmids (Marbouty et al., 2014; Press et al., 2017; Stalder et al., 2019) and phage-host infection spectrum (Marbouty et al., 2017). These studies suggest that meta3C and similar approaches hold the potential to 1) accurately bin genomes and episomal DNA molecules and 2) assign episomal DNA molecules to their respective hosts. However, comprehensive, end-to-end computational pipelines to process raw meta3C datasets remain sparse (Marbouty et al., 2017; DeMaere and Darling, 2019). Most analyses so far have focused on single mock communities, and quantifiable metrics are lacking to see how meta3C-like approaches truly compare—and possibly complement—traditional binning methods, notably regarding the quality, completeness, and accuracy of retrieved bins.

To address this need, we developed MetaTOR (Metagenomic Tridimensional Organisation–based Reassembly), a lean and scalable tool to investigate single or multiple proximity-ligation (i.e., 3C or Hi-C libraries) metagenomic experiments, from raw 3C reads and assembly to bins. MetaTOR was applied on 20 meta3C libraries of mouse gut samples collected over time. This first dynamic meta3C study allowed us to reconstruct dozens of complete genome sequences, and to compare the genomic bins recovered using MetaTOR with bins generated by binning software MetaBAT (Kang et al., 2015) and CONCOCT (Alneberg et al., 2014). MetaTOR compared favorably with respect to the number of high-quality genomes recovered (Bowers et al., 2017) and the amount of binned sequences. In addition, 3C-based binning was less dependent on the quality of the metagenome assembly (in terms of fragmentation—i.e., contigs’ mean size, N50). Overall, MetaTOR is a robust tool to process proximity-ligation sequencing data, regardless the number of samples processed.

## Materials and Methods

### Feces Sampling and meta3C Library Generation

The feces of three groups of two mice were sampled over 20 days as follows: days 2, 5, and 9 for cage n°1; days 2, 4, 5, 6, 7, 9, 10, 12, and 16 for cage n°2; and days 2, 5, 6, 7, 9, 11, 12, and 16 for cage n°3 (**Supplementary Figure 1**). The samples were immediately cross-linked after sampling in 30 ml of 1X tris-EDTA buffer supplemented with 3% formaldehyde (final concentration), for 1 h at room temperature with agitation. Formaldehyde was quenched by adding 10 ml of 2.5 M glycine during 20 min at room temperature with moderate agitation. Samples were then recovered by centrifugation, and pellets were stored at −80°C until processing. The libraries were then prepared and sequenced using pair-end (PE) Illumina sequencing (2 × 75 bp NextSeq) as described (Marbouty et al., 2014; Foutel-Rodier et al., 2018).

### Read Processing and Assembly

The first 10 bp of each read correspond to custom-made amplification primers allowing to remove PCR duplicates from the read pool (Marbouty et al., 2015). Those 10 bp were removed afterwards, and the resulting 65-pb sequences were filtered and trimmed using cutadapt (Martin, 2011). Quality was controlled with FastQC, and a total of 813 million PE reads were kept in total (over the 20 samples). Reads from libraries sampled from 1) cage 3 at day 2, 2) cage 3 with all samples, and 3) all cages with all samples were then used to perform three independent assemblies using MEGAHIT v1.1.1.2 (Li et al., 2015) with default parameters. Contigs under 500 bp were discarded from further analyses.

### Assemblies Analysis

Contigs from the three assemblies were analyzed with the MG-RAST pipelines (Meyer et al., 2008). The metagenomics RAST server allows automated annotations of complete or draft microbial genomes and provides information on phylogenetic and functional classification of the contigs. It also provides an alpha diversity measurement of the assembly.

### Alignment Step and Network Generation

Filtered reads were aligned independently in single-end mode using Bowtie2 v2.2.9 (option—very-sensitive-local) against one of the assemblies. For each sample, both alignment files were sorted and merged using the SAMtools and pysam libraries. Ambiguous alignments and alignments with mapping quality under 20 were discarded. All pairs of reads for which both reads aligned unambiguously on two different contigs were kept to generate the network. Contigs were considered as nodes, and the values of the edges (i.e., the weight) of the network were determined by counting the number of non-ambiguous alignments bridging the corresponding two contigs. Normalization was computed by dividing the edge value by the geometric mean of the nodes’ coverage (i.e., contigs’ coverage). Contig coverage was calculated using MetaBAT 1 v0.32.5 script: jgi_summarize_bam_contig_depths with a contig size limit of 500 bp for every set of reads.

### Louvain Clustering

We showed before that the updated implementation of the Louvain community method provided in (Blondel et al., 2008) was a promising approach to identify subnetworks of contigs in the meta3C network that display enriched contacts between themselves (Marbouty et al., 2014). The Louvain algorithm was run 400 times on each network, using the classical Newman-Girvan criterion. Nodes that systematically clustered together for each of the first 100 iterations were pooled together in core communities (CCs), as described previously (Marbouty et al., 2017).

### CCs Validation/Evaluation and Taxonomic Annotation

CCs above 500 kb were evaluated for completeness and contamination using CheckM version 1.0.7 (Parks et al., 2015). A CC was validated as a bin if its contamination rate range under 10%. CheckM was also used to assign taxa, at the class level, to validated bins using the *lineage* workflow.

### MAGs Evaluation

Validated bins were further evaluated following the standards to classify MAGs as high quality, medium quality, or low quality (Bowers et al., 2017). tRNA were searched with tRNAscan-SE 2.0 (Lowe and Eddy, 1997) (option -B). 16S and 23S rRNAs were searched using METAXA2 (Bengtsson-Palme et al., 2015)(options: -g SSU and -g LSU, respectively). We used RNAmmer-1.2 (Lagesen et al., 2007) (options: -S bacteria -m tsu) to look for 5S RNA. Bins were considered high-quality draft if they had 18 or more different tRNAs and at least one of each rRNA gene.

### Recursive Louvain Clustering

Partially complete CCs (> 70% completion) with contamination levels upper than 10% were selected for recursive binning. Briefly, the partition step was re-run 10 times on these contaminated CCs (i.e., on their corresponding sub-network), yielding groups of smaller CC (i.e., sub-CCs) which were then re-processed in the binning step to assess for their quality.

### Pipeline Comparison

CONCOCT v1.0.0 (Alneberg et al., 2014) and MetaBAT 1 v0.32.5 (Kang et al., 2015) were run on the same set of reads and assemblies, using the different time samples for differential coverage. Resulting bins above 500 kb were retrieved and compared with MetaTOR’s for completeness and contamination using CheckM. CONCOCT was run with the following parameters –r 65 -s 100. MetaBAT 1 was run with default parameters.

## Results

### Algorithmic Principles Underneath the MetaTOR Pipeline

**Figure 1:**
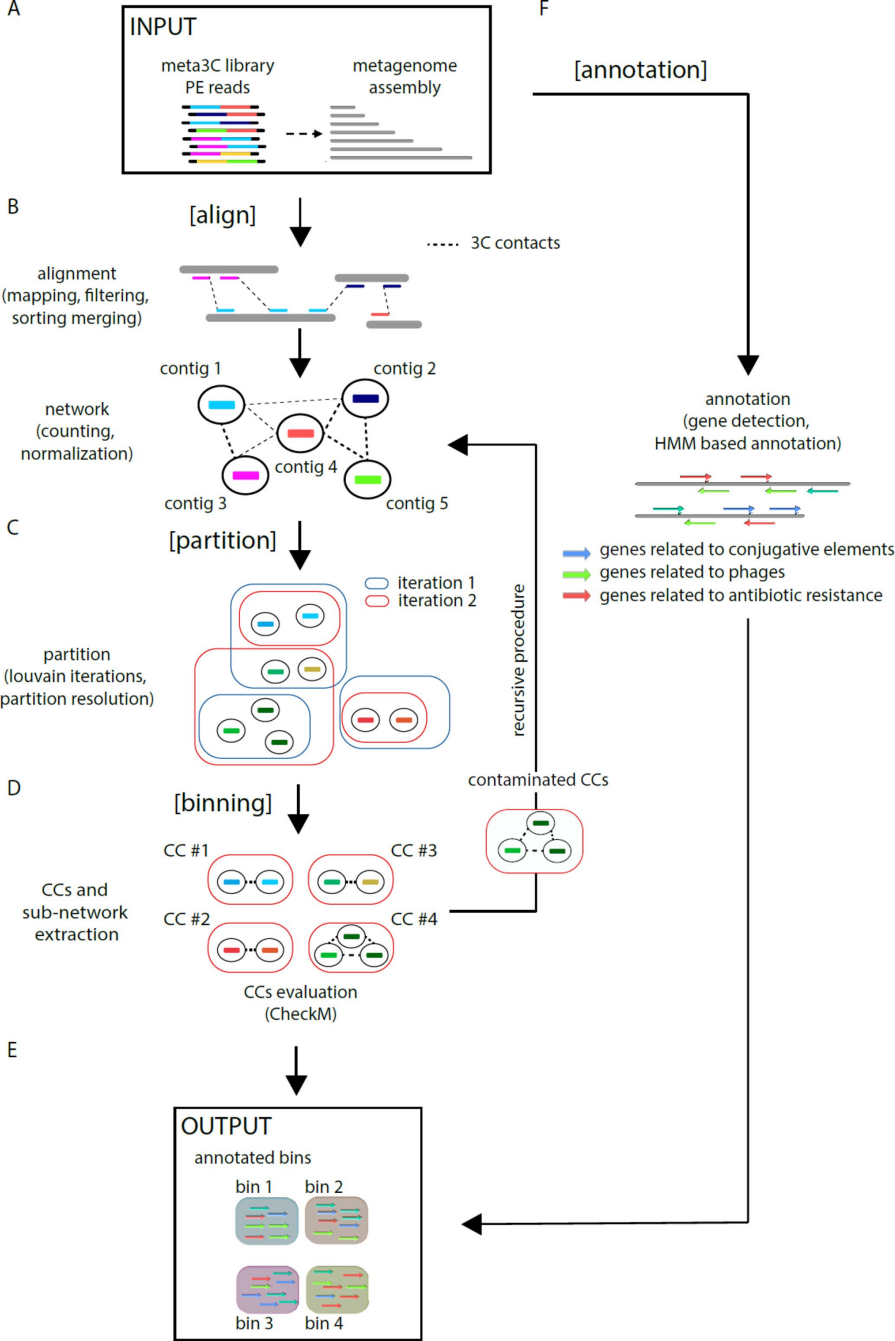
MetaTOR pipeline. Schematic representation of the MetaTOR pipeline. **(A)** MetaTOR is initialized with an assembly and a set of 3C/Hi-C PE reads. **(B)** [Align] will align, sort, and merge reads to deliver a network of contig interactions. **(C)** [Partition] will deconvolve the previously defined network using a Louvain iterative procedure and **(D)** [Binning] will retrieve CCs (FASTA file and corresponding sub-network) from selected partition to evaluate them using CheckM. At this step, it is possible to perform a recursive procedure on selected CCs to split them further into sub-CCs. **(F)** [Annotation] is an optional step that use HMM models to provide final annotations. **(E)** The final output of the pipeline is a set of annotated bins.

MetaTOR (https://github.com/koszullab/metaTOR) aims at providing the most accurate overview of genome content of a population, starting from as little as one meta3C library, while taking full advantage of additional libraries if available. It’s structured around four main steps: alignment, partition, annotation, and binning (**Figure 1**). MetaTOR was purposely designed to maintain a high level of modularity and flexibility, so that users can supply their own intermediary inputs and tweak parameters to their liking at every step. This can save both time and resources. If starting from the raw data, all needed is the meta3C PE files and an assembly of the microbial community obtained either directly from the meta3C reads (as described in this work and in Marbouty et al., 2014; Marbouty et al., 2017) or from a DNA library generated independently (**Figure 1A**).

- **[Align] **(**Figure 1B**): First, meta3C reads are aligned independently along the contigs of the metagenome assembly using Bowtie2 (as aligners tend to leave out far-off alignments when run in PE mode). Contigs are then sorted, filtered for mapping quality, and merged into a global alignment file. The alignment is converted into a contact network stored in a plain text file [network.txt: column 1—node 1/column 2—node 2/column 3—weight] to facilitate further third-party analysis. In the network, each node represents one contig, and each edge (a.k.a. weight) represents the contact score found between two contigs. This step integrates variable parameters such as enforcing a lower size limits for contigs or a normalization step. Normalization of the network typically uses contig coverage, but other normalizations can be implemented as well.
- **[Partition] **(**Figure 1C**): An iterative Louvain procedure is applied on the network file to partition the network into groups of contigs that consistently cluster together, i.e., “see” each other’s in space more often than their neighbors’ (Blondel et al., 2008; Marbouty et al., 2014; Marbouty et al., 2017). These clusters or CC constitute the matrix of the metagenomic binning. The number of iterations is a free parameter of the pipeline and can be set by the user. However, we noted that the number of CC stabilizes after a while with small oscillations around a fixed value, and therefore recommend enough cycles to reach that threshold.
- **[Binning] **(**Figure 1D**) CCs are then extracted (FASTA files) and their gene content assessed for completeness and contamination using CheckM (Parks et al., 2015). In parallel, the pipeline extracts sub-networks for each CC (i.e., network between the corresponding contigs). Extraction of each sub-network allows the user to perform, if needed, a recursive procedure at this step on the defined contig group (i.e., CCs) (see **Figure 1**—“recursive procedure”). Indeed, some CCs exhibit both a high completion rate and a high contamination levels suggesting that they contain more than one genome. By applying the partition step only on their corresponding sub-network, it becomes possible to sub-partition using the Louvain algorithm these CCs into smaller ones (i.e., sub-CCs). This step typically breaks down the most contaminated CCs into smaller, low-contaminated sub-CCs. The retrieved sub-CCs can also be evaluated using CheckM and validated as bins.
- **[Annotation] **(**Figure 1F**): Gene prediction is performed using Prodigal (Hyatt et al., 2010), and genes of interest are detected using HMM models publicly available (Albertsen et al., 2013; Guglielmini et al., 2014; Grazziotin et al., 2017). However, this step is independent from the others, and any annotation tool can be applied instead.

MetaTOR generates a set of annotated metagenomics bins and their corresponding FASTA sequences (in addition to the contact network) (**Figure 1E**).

### Construction of meta3C Libraries and Generation of Metagenome Assemblies

To validate and compare the pipeline to classical metagenomic binning algorithms, we investigated the gut microbiota of various mice using meta3C libraries. Feces were sampled from three groups of two mice from the Institut Pasteur animal facility, over 20 days (Materials and Methods) (**Supplementary Figure 1**). Twenty meta3C libraries (three from cage n°1, nine from cage n°2, and eight from cage n°3) were then generated as described (Marbouty et al., 2017) (*Materials and Methods*) using HpaII as restriction enzyme. Libraries were sequenced using PE Illumina 2x75 bp Kits (**Table 1**) (NCBI BioProject PRJNA542645). After trimming and quality filtering, between 25 and 100 million PE reads were recovered for each of the samples (∼813 million PE reads total).

**Table 1:** Meta3C libraries constructed and sequenced.

Sample	Raw paired-end reads
Cage1-day1	79 868 626
Cage1-day2	38 728 350
Cage1-day3	33 173 429
Cage2-day1	40 380 356
Cage2-day2	62 424 123
Cage2-day3	31 436 086
Cage2-day4	34 124 320
Cage2-day5	48 472 570
Cage2-day6	36 129 310
Cage2-day7	32 608 370
Cage2-day8	43 473 731
Cage2-day9	67 768 796
Cage3-day1	108 114 353
Cage3-day2	39 719 377
Cage3-day3	37 792 067
Cage3-day4	36 805 550
Cage3-day5	34 529 306
Cage3-day6	59 092 136
Cage3-day7	28 833 461
Cage3-day8	30 521 091

Meta3C sequences can be directly used to generate a *de novo* assembly without notable increase of false/chimeric contigs (Marbouty et al., 2014). Three assemblies (1, 2, and 3) using reads collected from cage 3/day 2, cage 3/all samples, and all cages/all samples, respectively, were generated using MEGAHIT (Li et al., 2015) (*Materials and Methods*). After discarding contigs under 500 bp, the three assemblies resulted in 61,600, 167,810, and 237,868 contigs for a cumulated size of 146, 475, and 763 Mb, respectively (**Table 2**). These assemblies and their corresponding set of reads were used to test the binning pipelines MetaTOR, MetaBAT, and CONCOCT, and their output (*Material and Methods*). The number of species present in the total assembly (n°3) was estimated using MG Rast and the alpha diversity provided for the assembly (Meyer et al., 2008) (*Material and Methods*). In total, 268 bacterial genomes are predicted to be present in the global assembly.

**Table 2:** Assembly metrics. Only the metrics concerning assemblies filtered for the contigs above 500 bp are shown.

	PE reads (filtered)	Total size (contigs > 500 bp)	Contigs > 500 bp	N50 (contigs > 500 bp)
Assembly #1 (cage 3—day 2)	100,258,683	146,319,508 bp	61,666	6,176 bp
Assembly #2 (cage 3—samples x 8)	330,324,521	475,681,220 bp	167,810	7,578 bp
Assembly #3 (samples x 20)	813,376,239	763,455,888 bp	237,868	12,339 bp

### Binning of Metagenomes Using MetaTOR

**Figure 2:**
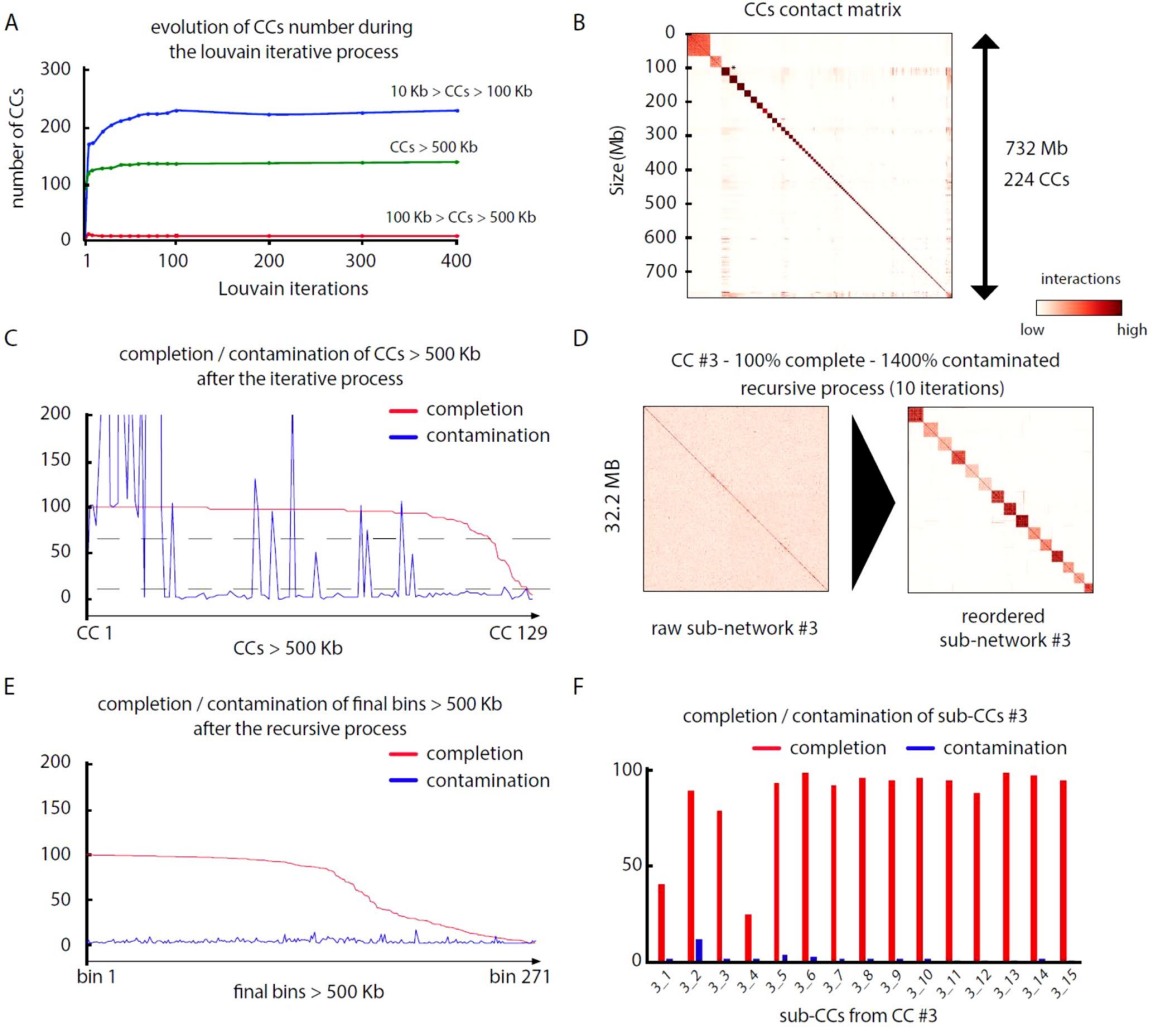
MetaTOR partitioning of a complex microbial community. **(A)** Evolution of the number of CCs, ordered by size categories, during 400 Louvain iterations for assembly n°3 (20 samples). Color represents the amount of DNA in a given CC. Blue: 10 to 100 kb. Red: 100 to 500 kb. Green: > 500 kb. **(B)** Contact matrix encompassing the 224 largest CCs ordered by size, after 100 Louvain iterations (1 pixel = 200 kb). Y-axis: cumulated DNA size. **(C)** Completion (red) and contamination (blue) of the 129 CCs containing more than 500 kb after 100 Louvain iterations. Dashed lines: thresholds used to process CCs through a recursive procedure (completion threshold: upper 70%; contamination threshold: upper 10%). **(D)** Contact map of a highly contaminated CC (CC #3—100% complete—1,400% contaminated) before (left) and after (right) the recursive procedure (10 iterations; 1 pixel: 20 kb). Left map: contigs are ordered by size. Right map: sub-CCs are ordered by size. **(E)** Completion and contamination of the 269 CCs and sub-CCs bigger than 500 kb defined after the whole procedure. Red: completion. Blue: contamination. **(F)** Completion (red) and contamination (blue) levels of the sub-CCs retrieved from the original CC #3 after recursive procedure (10 iterations).

Pairs of meta3C reads were aligned independently on their respective assembly to identify those for which both reads aligned on different contigs (parameters: MQT = 20; contig size limit = 500 bp). Normalized contact scores between contigs where computed by dividing the number of pairs bridging two contigs by the square root of the product of each contig coverage. For each assembly, this step generates a network of weighted connections between contigs (**Table 3**). Each network was subsequently partitioned into CCs through iterative Louvain partitioning. After ∼100 cycles, the number of large CCs (>500 kb) reaches a plateau for the three networks (**Figure 2A**). Contacts between CCs appear low, suggesting that contigs interacting preferentially with each other’s were successfully pooled together (**Figure 2B**).

**Table 3:** Network features.

	PE reads (filtered)	Mapped PE reads	Intercontig interactions	Weighted interactions
Assembly #1	100,258,683	67,994,798	6,457,842	1,322,003
Assembly #2	330,324,521	215,768,714	30,206,795	8,505,609
Assembly #3	813,376,239	541,384,131	96,546,376	77,577,924

We analyzed, using CheckM (Parks et al., 2015), the gene content of the 17, 33, and 125 CCs > 500 kb from assemblies 1, 2, and 3, respectively. Most CCs showed completion and contamination levels above 80% and under 10%, respectively (**Figure 2C**), suggesting that they contain near-complete bacterial genomes. Those CCs were annotated as valid bins or MAGs. However, a subset of CCs displayed high contamination rate, from 10% to more than 1,000% while showing high 70/80% completion levels as well (4, 24, and 25 CCs for assemblies 1, 2, and 3, respectively) (**Figure 2C**). We suspected that these high contamination rates reflected the pooling of DNA contigs belonging to related species sharing conserved/similar sequences. We therefore applied on these CCs an extra recursive procedure consisting of processing them with 10 Louvain clustering steps. This generated sub-networks or sub-CCs (**Figure 2D**) that often display high-quality signatures of bacterial genomes, showing that indeed the large, contaminated CCs correspond to mixes of near-complete bacterial genomes (**Figure 2F**). These sub-CCs also often belonged to the same taxonomic group, suggesting that indeed sequence homology between closely related species bridged these contigs together. A focus on assembly #3 shows that the computation generated 1,001 bins > 10 kb corresponding to 724 Mb, among which 686 Mb (95%), was included within 271 bins larger than 500 kb (**Figure 2E**). This number can be compared to the 268 genomes predicted to be present in the assembly (above; *Materials and Methods*). The average completion and contamination levels of these CCs are 65.8% and 2.4%, respectively (to compare with 88.4% and 61.4% if the recursive procedure was not applied). MAG evaluation was performed (Bowers et al., 2017), resulting in 82 high-quality (< 5% contamination, > 90% completion and presence of the 23S, 16S, and 5S rRNA genes and at least 18 tRNAs), 87 medium-quality (< 10% contamination and > = 50% completion), and 96 low-quality MAGs (< 10% contamination and < 50% completion) (**Table 4**) (other MAGs display more than 10% of contamination; **Supplementary Table 1**).

**Table 4:** Comparison of MetaTOR, CONCOCT, and MetaBAT results.

		Assembly #1 (148 Mb)		Assembly #2 (483 Mb)		Assembly #3 (763 Mb)	
		Nb	Size (bp)	Nb	Size (bp)	Nb	Size (bp)
Metator	10 kb < bins < 100 kb	284	7,537,821	807	21,139,528	617	15,175,457
	100 kb < bins < 500 kb	43	11,319,827	144	30,749,287	106	22,963,515
	Bins > 500 kb	56	119,111,306	183	399,972,204	271	685,955,810
	Low-quality MAGs	31	36,042,593	97	107,071,523	96	128,486,895
	Medium-quality MAGs	16	47,397,754	39	131,055,387	87	285,670,443
	High-quality MAGs	9	35,670,959	41	140,967,746	82	259,541,396
MetaBAT	10 kb < bins < 100 kb	0	0	0	0	0	0
	100 kb < bins < 500 kb	18	5,703,905	55	17,583,986	65	24,087,225
	Bins > 500 kb	36	82,290,484	126	284,973,235	172	420,081,339
	Low-quality MAGs	14	12,478,196	44	52,797,176	95	36,277,628
	Medium-quality MAGs	21	61,439,633	73	202,719,703	143	322,230,178
	High-quality MAGs	0	0	3	5,488,345	22	58,276,800
CONCOCT	10 kb < bins < 100 kb	11	432,808	25	1,040,872	24	1,122,733
	100 kb < bins < 500 kb	7	1,351,308	23	6,275,583	6	5,193,580
	Bins > 500 kb	29	120,778,514	126	412,598,588	195	673,338,423
	Low-quality MAGs	8	17,152,380	41	76,579,222	42	70,748,222
	Medium-quality MAGs	11	25,303,368	49	134,612,509	114	358,231,099
	High-quality MAGs	0	0	11	49,146,272	12	47,807,957

### Comparison With Hybrid Binning Algorithms

**Figure 3:**
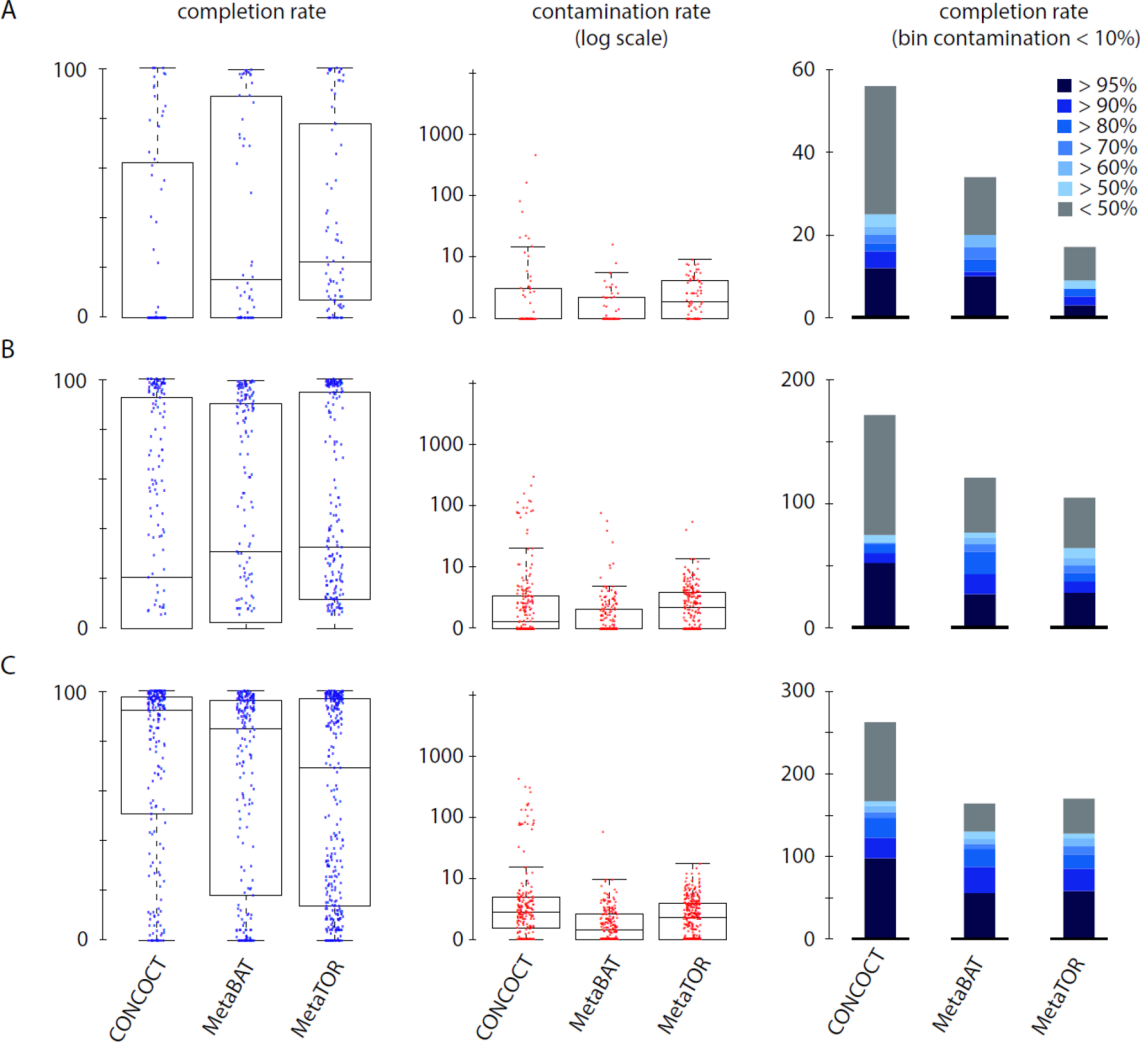
Comparison of MetaTOR, MetaBAT, and CONCOCT. CheckM output comparison for the three binning methods applied on the three assemblies tested in this work. **(A)** Assembly 1 (one meta3C library). **(B)** Assembly 2 (eight libraries). **(C)** Assembly 3 (20 libraries). Box plot for completion (left) and contamination (middle) and histogram of retrieved MAGs (right) are presented for the three binning methods. Only MAGs over 500 kb and harboring less than 10% of contamination are analyzed.

**Figure 4:**
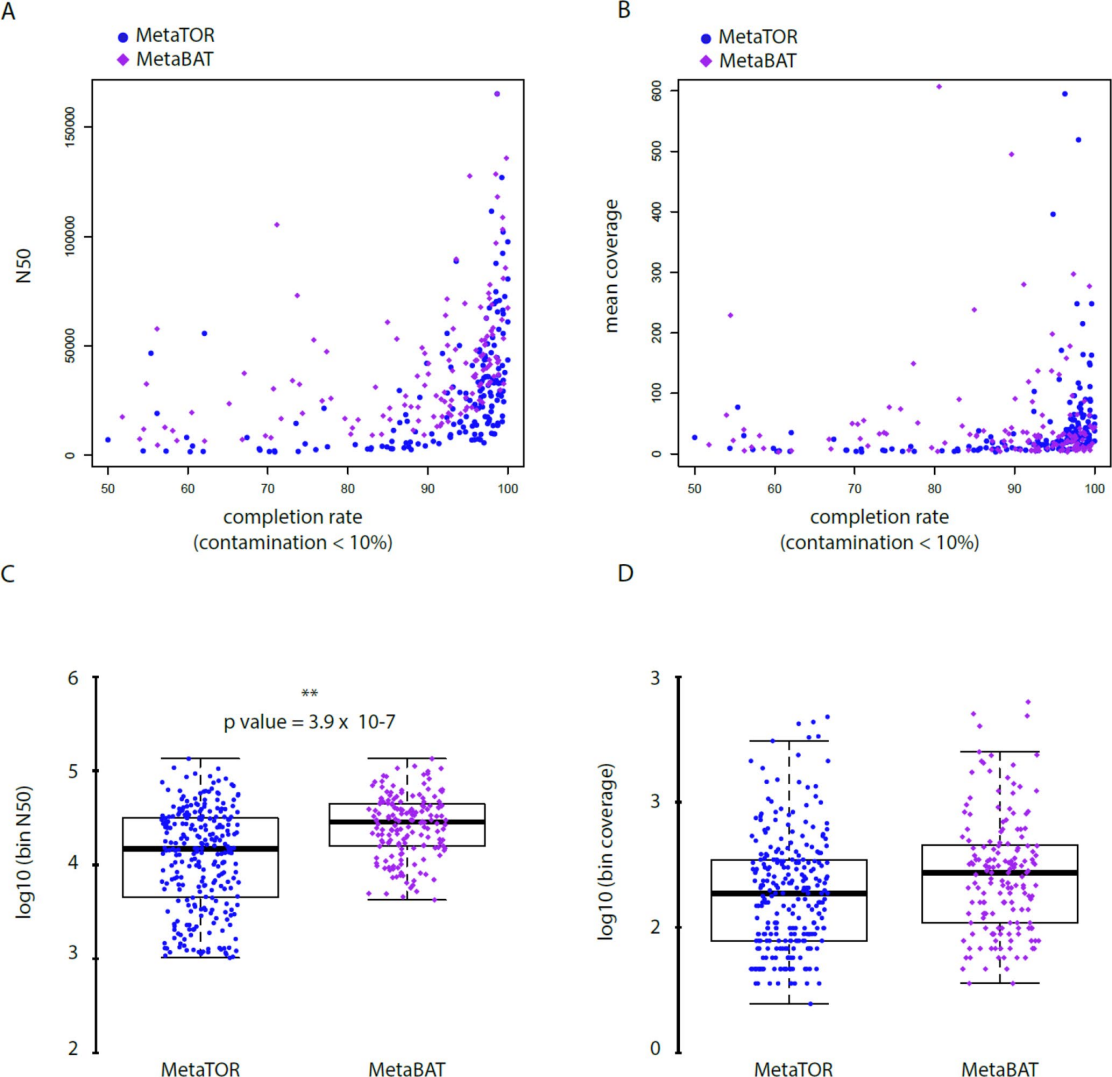
Statistics of low contaminated reconstructed bins. **(A–B)** Correlation between completion rate and N50 **(A)** or mean coverage **(B)** for bins with a contamination rate below 10%. Blue circles = MetaTOR bins. Purple diamonds = MetaBAT bins. ) **(C–D)** Box plot for N50 **(C)** and mean coverage **(D)** of retrieved bins with a contamination rate below 10% are presented for MetaTOR (blue circles) and MetaBAT (purple diamonds). A t-test shows a clear difference between distribution of bins’ N50 for the two software (**C**—p-value = 3.9 x 10^-7^).

To evaluate how MetaTOR compares to existing binning approaches, we ran MetaBAT (v.1; Kang et al., 2015) and CONCOCT (Alneberg et al., 2014) on assemblies #1, #2, and #3 using the same filtered PE reads, allowing each pipeline to take advantage of the information from differential coverage across the independent experiments. The metric used to assess the efficiency of the three programs is their CheckM output (i.e., levels of completion and contamination) and the number of high-/medium-/low-quality MAGs (**Figure 3** and **Table 3**). For the three assemblies, MetaTOR retrieved 9, 41, and 82 high-quality MAGs, compared to 0, 3, and 22 with MetaBAT and 0, 11, and 12 with CONCOCT. MetaTOR also retrieved more bins exhibiting a high completion/low completion rate (90–10%) (**Figure 3**). The mean completion and contamination rates of bins characterized by MetaBAT using the 20 libraries were slightly better (respectively, 74% and 1.7%) than the ones obtained using MetaTOR (respectively, 65.8% and 2.4%) (**Figure 3**), but this could be due to the greater number of bins (>500 kb) obtained using MetaTOR (MetaBAT = 172; MetaTOR = 271) (**Table 4**). To compare further the output of MetaTOR and MetaBAT and their ability to reconstruct genomes from different phyla, we analyzed the taxonomic annotations of assembly #3 with the taxonomy of all the bins above 500 Kb retrieved for this assembly (**Supplementary Figure 2**). The bins generated by both softwares were highly consistent with the assembly annotation suggesting that they do not present particular taxonomic bias in their binning process. To evaluate MAGs, 16S and 23S rRNA were searched in assembly #3 using METAXA2 (Bengtsson-Palme et al., 2015). A total of 507 23S rNRA and 304 16S rRNA were found but only 213 and 143, respectively, were located on contigs longer than 1 kb. As CONCOCT and MetaBAT only use contigs upper 1 kb, this severely decrease the amount of potential rRNA found in their bins and could explain why they were only able to bin very few high-quality drafts according to MiMAG standards (rRNA were very often the limiting factor to classify bins in that category) (Bowers et al., 2017). We then wonder if our method, which can bin contigs regardless of their size, shows better results concerning low-covered and/or highly fragmented genomes. We looked at the relation between completion for bins with a contamination rate lower than 10% and assembly statistics for those bins (**Figure 4**). Whereas we could not see clear differences between MetaBAT and MetaTOR when we look at the bins’ mean coverage (**Figure 4B–D**), it appears clearly that the contigs’ fragmentation is a limiting factor for MetaBAT as observed when we plotted the completion rate in function of the N50 (**Figure 4A–C**). These observations suggest that MetaTOR is able to accurately bin relatively fragmented genomes and correctly assign contigs smaller than 1 kb.

## Discussion

We previously showed that a blind analysis of meta3C/proximity-ligation reads generated from controlled and unknown, complex mixes of species could be used to characterize efficiently their genomes (Marbouty et al., 2014; Marbouty et al., 2017). In the present work, we extend our original approach by developing a scalable computational pipeline, MetaTOR, and applying it on multiple samples of meta3C gut microbiota libraries. Compared to binning programs MetaBAT and CONCOCT, MetaTOR was able to retrieve more high-quality MAGs from highly fragmented assemblies. This work shows that physical collisions between DNA sequences represent an objective, quantitative measure to cluster these molecules together. This approach could therefore nicely complement or replace popular approaches that exploit sequence composition correlations or abundance co-variation. This remains true even when 20 independent experiments were used, highlighting the interest to include room for some meta3C experiments in future metagenomics projects, regardless of the number of planed libraries. Meta3C-like methods have only been applied to microbial rich samples so far (mice and human gut, cow rumen, sewage) (Marbouty et al., 2017; Stewart et al., 2018; Stalder et al., 2019) and still need to be improved in order to be applied to sample with low concentration of microorganisms. The time needed to generate a meta3C library is 3 days, and up to 16 libraries can be generated in parallel (Foutel-Rodier et al., 2018). It is also likely that commercial kits will be available relatively soon, which will boost the amenability of the approach. The cost of a single library is estimated to ∼50€ (not including processing and sequencing of the library). Therefore, we believe this approach is well suited for a variety of metagenomics projects.

A limitation of the present work consists in the size of the reads sequences, 65 bp, whereas most metagenomics studies sequence longer reads (150 bp). This is probably a disadvantage for the two binning programs we tested as the assembly quality is technically lower than what it would have been if computed with longer reads. On the other hand, one could also argue that meta3C/MetaTOR can therefore be performed using cheaper, short-read sequencing approaches and still provide good results. However, more tests are needed to fully characterize the influence of assembly quality on the different programs in light of MetaTOR results.

To improve the assembly, regardless of the read length, it is also possible to apply the approach used in Marbouty et al. (2017), which consists in mapping back the total reads (including ambiguous ones originally discarded) back to contigs of one bin. These reads are then used to generate a new assembly for each individual bin. Although time consuming, we showed that this approach improved the assembly statistics of each bin (Marbouty et al., 2017).

The large networks derived from different meta3C libraries contain several highly connected sub-networks poorly connected to each other. Highly modular networks such as those are known to be well-suited for community detection algorithm like Louvain (Blondel et al., 2008). Moreover, the “iterative Louvain” procedure allows us to identify sets of sequences that contact each other. However, there are limits to the current iterative Louvain implementation. First, all modularity optimization algorithms tend to over-cluster nodes when the network reaches a certain size threshold, regardless of the underlying patterns. This well-documented property is known as the “resolution limit” (Fortunato and Barthélemy, 2007). However, it can be sidestepped by running the partitioning process recursively on the network corresponding to the studied sub-network. Since it should be comparatively small and under the scale at which the aforementioned limit becomes visible, the clusters found inside will separate again and yield bins as normal. The recursive procedure applied in the present work appears as highly effective with a clear increase in the number of high-quality MAGs retrieved.

A second limit comes from the stringent definition of CCs that consist of sequences that always, systematically cluster together. As a result, a single “jump” of a contig out of a cluster during one of the iterations will lead to its exclusion from the final CCs. While this allows contamination reduction, a number of meaningful sequences could still incidentally be excluded from the bin. Indeed, mobile or repeated elements (e.g., phage, prophages, or plasmids) shared by different species will likely be excluded from their corresponding CCs. This limitation can be overcome *a posteriori* as follows. First, annotation pipelines such as VirSorter (Roux et al., 2015) or PlasFlow (Krawczyk et al., 2018) allows to identify contigs carrying such sequences. Second, the bacterial hosts of these contigs can be inferred using the contact network as described in (Marbouty et al., 2017), and/or with the help of the Louvain clustering score (computed from the iterative procedure, and corresponding to the number of times two CCs are grouped together). A detailed analysis of overlapping communities would be very useful in the future to study such associations and bring a new tool in the study of interactions between genomic entities in microbial communities.

Our pipeline is flexible: although it was developed to take advantage of the Louvain algorithm, other clustering algorithms yielding nondeterministic community identifiers (e.g., a community detection algorithm with a different modularity) can be used instead with no side effects on the rest of the pipeline.

Proximity-ligation assays were originally developed to capture the 3D folding of microbial or mammalian chromosomes (Dekker et al., 2002; Lieberman-Aiden et al., 2009). Derivative applications of these techniques were developed and applied to solve or improve genomics techniques such as chromosome-level scaffolding (Kaplan and Dekker, 2013; Burton et al., 2013; Marie-Nelly et al., 2014), haplotype reconstruction (Selvaraj et al., 2013), or centromere annotation (Marie-Nelly et al., 2014). Haplotype phasing is a particularly interesting development to combine with metagenomics data since strains from the same species remain challenging to characterize. This requires both an improvement in meta3C like capture yield to increase the resolution in coverage of the contigs, as well as the integration of computational haplotype phasing programs.

## Data Availability

The datasets generated for this study can be found on SRA database: BioProject PRJNA542645.

## Ethics Statement

Animal experimentation: The Institut Pasteur ethics organism (CETEA) approved all the experiments performed on mice (Project dha170005).

## Author Contributions

MM and RK conceived the study. LB, TFR and MM wrote the pipeline MetaTOR. MM, TFR, and AT performed the experiments. LB, TFR, MM, and RK analyzed and interpreted the data. LB, TFR, MM, and RK wrote the manuscript.

## Funding

LB is supported by an AMX fellowship from the French Ministry of Higher Education, Research and Innovation. TFR is supported by an ENS fellowship by the French Ministry of Higher Education, Research and Innovation. This research was supported by funding to RK from the European Research Council under the Horizon 2020 Program (ERC grant agreement 260822) and from the Agence Nationale pour la Recherche (JPI-EC-AMR STARCS ANR-16-JPEC-0003-05).

## Conflict of Interest Statement

The authors declare that the research was conducted in the absence of any commercial or financial relationships that could be construed as a potential conflict of interest.
