# Supplementary material for: MetaTOR: A Computational Pipeline to Recover High-Quality Metagenomic Bins From Mammalian Gut Proximity-Ligation (meta3C) Libraries
Source: Front Genet. 2019 Aug 20;10:753. doi: 10.3389/fgene.2019.00753 (PMC6710406; doi:10.3389/fgene.2019.00753)
Supplement: Supplementary file 1 [file DataSheet_1.pdf]

Supplementary Material

MetaTOR: a computational pipeline to recover high-quality metagenomics bins from mammalian gut proximity-ligation (meta3C) libraries

Lyam Baudry[1,2,3†], Théo Foutel-Rodier[1,2,3†], Agnès Thierry[1,2], Romain Koszul[1,2,*] and Martial Marbouty[1,2,*]

1 Supplementary Figures and Tables

1.1 Supplementary Figures

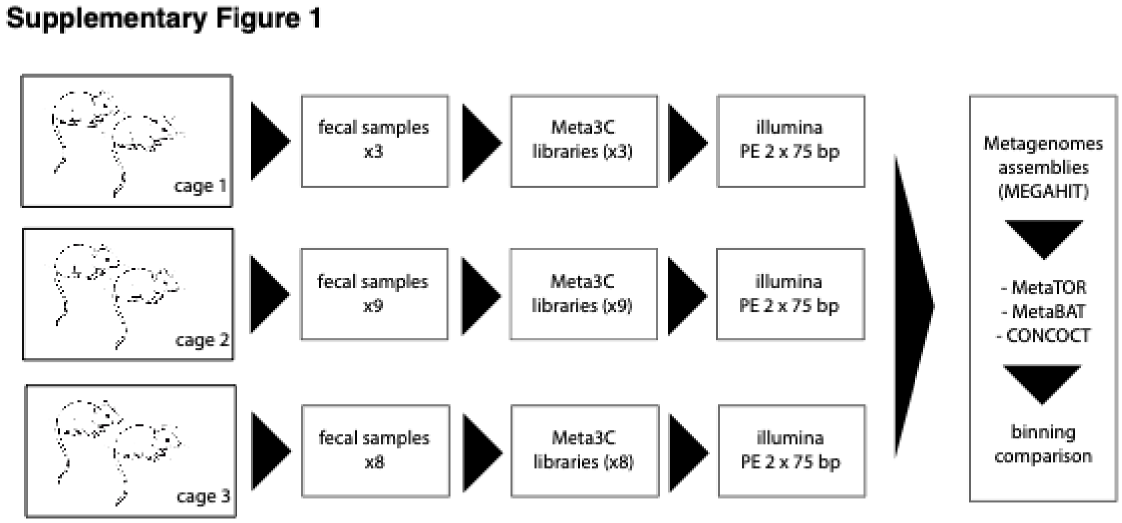

Supplementary Figure 1. Experimental design

Three groups of two mice were sampled during twenty days as follow: day 2, 5 and 9 for the cage n°1; day 2, 4, 5, 6, 7, 9, 10, 12, 16 for the cage n°2; day 2, 5, 6, 7, 9, 11, 12, 16 for the cage n°3. Samples were then processed for meta3C sequencing. The resulting sequences were used to generate *de novo* assemblies and test the different binning methods.

Supplementary Figure 2

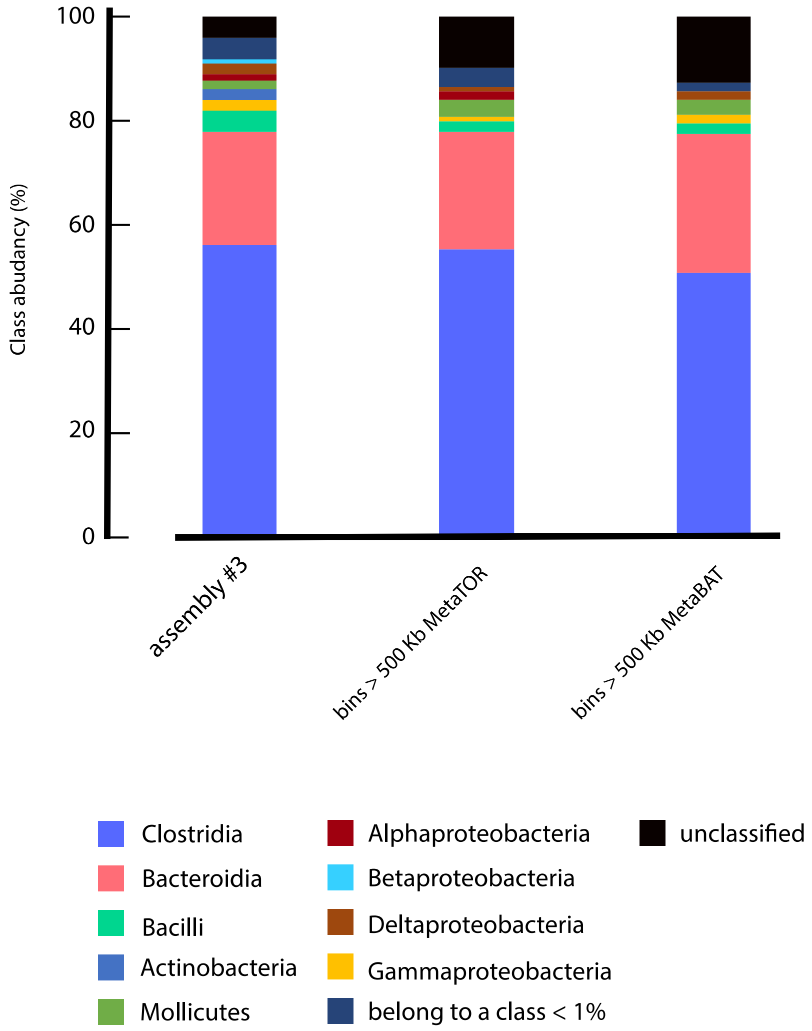

Supplementary Figure 2. Class level composition of assembly #3 and bins (>500 Kb) from MetaTOR and MetaBAT.

All taxonomic groups representing less than 1% of sequences (contigs or bins) were grouped into "belong to a class < 1%". Any taxonomic group unable to be assigned to a kingdom (bacteria or archaea) was grouped into "unclassified".

Supplementary Figure 3

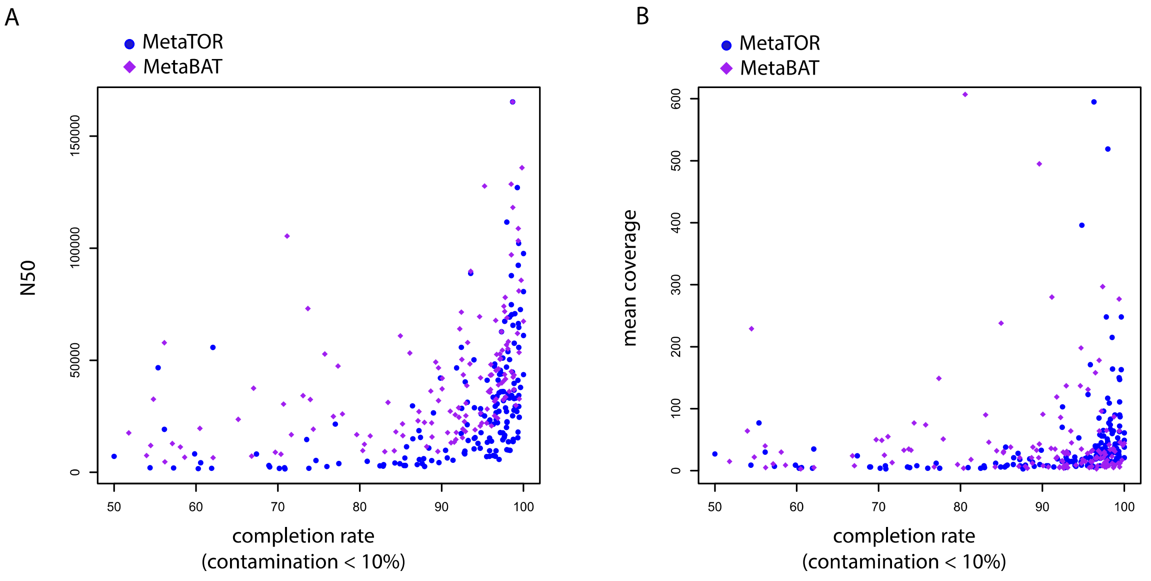

Supplementary Figure 3. Correlation between completion rate and bins properties

A-B. Correlation between completion rate and N50 (A) or mean coverage (B) for bins with a contamination rate below 10%. Blue circle = MetaTOR bins. Purple diamond = MetaBAT bins.

1.2 Supplementary Tables

Supplementary Table 1: completion / contamination and assembly statistics of the different bins reconstructed using MetaTOR.

bin ID	Completion (%)	Contamination (%)	Genome size (bp)	Number of contigs	N50 (bp)	mean contigs size (bp)	longest contig (bp)
bin_39	100.00	4.55	4145194	355	43597	11676	167487
bin_27	100.00	0.00	4582350	386	61027	11871	196515
bin_155	100.00	0.48	1956233	122	97607	16034	218330
bin_113	100.00	0.04	2487128	129	80619	19280	201368
bin_69	99.63	1.48	3422137	452	37864	7571	104329
bin_5	99.62	1.41	6806542	425	72595	16015	232119
bin_116	99.48	1.92	2449015	411	24572	5958	134569
bin_84	99.43	0.94	3053587	285	64664	10714	238659
bin_111	99.43	0.40	2506347	246	34319	10188	116698
bin_50	99.42	0.10	3798412	306	55722	12413	180635
bin_21	99.42	1.51	4916183	789	29370	6230	110755
bin_151	99.41	0.35	2012592	162	102105	12423	234280
bin_30	99.37	1.34	4429971	434	66371	10207	208940
bin_9	99.36	2.23	5740145	499	92347	11503	496357
bin_15	99.33	2.85	5141661	1194	17970	4306	110334
bin_3	99.32	2.75	7086290	832	41059	8517	199773
bin_8	99.31	5.48	6182655	1217	15426	5080	183186
bin_56	99.25	0.11	3691009	234	127004	15773	310695
bin_31	99.19	1.29	4412337	568	27123	7768	142491
bin_117	99.06	1.89	2433291	329	28684	7396	95516
bin_100	99.05	1.13	2724327	209	33100	13035	117710
bin_74	98.99	2.08	3322007	353	29449	9410	116382
bin_150	98.94	3.28	2038911	141	70784	14460	261084
bin_22	98.87	0.56	4839229	245	57609	19751	280706
bin_42	98.85	1.34	4019528	487	36676	8253	129931
bin_25	98.85	1.56	4737717	606	39679	7818	124301
bin_12	98.85	4.68	5405420	1146	32888	4716	169342
bin_11	98.83	2.96	5407594	917	27716	5897	91224
bin_112	98.75	2.74	2503265	344	14737	7276	51554
bin_14	98.73	3.80	5254560	1199	13740	4382	66252
bin_6	98.71	7.33	6461730	1489	13741	4339	63331
bin_26	98.71	2.53	4596695	505	65575	9102	250035
bin_91	98.67	2.92	2909925	320	25202	9093	81069
bin_157	98.67	0.22	1951218	152	165250	12836	457096

**bin_66**	98.66	0.50	3443291	659	44691	5225	264790
**bin_121**	98.56	4.01	2385882	167	70338	14286	299732
**bin_43**	98.55	0.12	3921113	293	34279	13382	145550
**bin_32**	98.52	2.74	4288715	518	87755	8279	291057
**bin_101**	98.51	8.55	2713635	264	74788	10278	261337
**bin_114**	98.36	3.04	2485838	631	9806	3939	40366
**bin_98**	98.30	0.01	2768987	154	69266	17980	156184
**bin_97**	98.30	0.72	2775067	229	50288	12118	169029
**bin_122**	98.28	2.59	2380393	296	33115	8041	129399
**bin_134**	98.16	3.18	2297045	520	13630	4417	48163
**bin_55**	98.14	3.56	3747241	949	9926	3948	54255
**bin_38**	98.10	0.00	4178182	332	53855	12584	175327
**bin_13**	98.10	2.53	5310772	888	34994	5980	168796
**bin_63**	97.99	2.77	3508420	698	22292	5026	156427
**bin_72**	97.99	0.00	3358957	565	22579	5945	115314
**bin_41**	97.97	4.61	4095733	1119	10102	3660	73784
**bin_92**	97.96	0.03	2909409	156	111597	18650	343448
**bin_96**	97.92	1.54	2793631	169	46960	16530	169551
**bin_95**	97.92	0.77	2814843	277	27152	10161	116503
**bin_62**	97.92	0.77	3522684	464	20009	7591	114294
**bin_58**	97.80	3.21	3614393	595	28758	6074	105139
**bin_107**	97.80	1.70	2575918	286	33288	9006	156008
**bin_85**	97.70	1.34	3049043	237	67378	12865	224602
**bin_7**	97.70	4.26	6211975	1188	16566	5228	115288
**bin_54**	97.65	2.94	3747733	770	15598	4867	78259
**bin_140**	97.55	0.97	2204843	175	32005	12599	108256
**bin_105**	97.55	1.23	2636724	211	36005	12496	193221
**bin_61**	97.47	0.65	3563061	332	50998	10732	120576
**bin_19**	97.47	2.43	4959815	998	17596	4969	79632
**bin_16**	97.47	2.08	5095463	1021	14521	4990	102524
**bin_29**	97.43	3.80	4467670	597	36022	7483	210984
**bin_115**	97.32	0.57	2485588	209	43945	11892	196034
**bin_145**	97.32	0.00	2088794	151	62711	13833	199392
**bin_80**	97.23	2.39	3116845	380	35966	8202	97332
**bin_70**	97.17	0.88	3386971	252	45537	13440	125386
**bin_135**	97.17	2.43	2292868	273	17722	8398	55209
**bin_57**	97.08	0.81	3682199	381	43692	9664	127288
**bin_18**	97.02	5.16	5050270	1509	5755	3346	61637
**bin_17**	97.01	5.46	5090563	604	35466	8428	155699
**bin_89**	97.00	2.45	2926234	272	27238	10758	88836
**bin_104**	96.90	0.48	2661293	245	41128	10862	152607
**bin_132**	96.83	3.47	2307673	221	22148	10441	54559
**bin_77**	96.79	1.78	3240951	276	30550	11742	114698
**bin_106**	96.77	1.45	2600911	229	33925	11357	107602
**bin_124**	96.67	0.78	2365390	285	23436	8299	63885

**bin_33**	96.64	7.52	4278153	1250	9584	3422	59164
**bin_123**	96.64	2.92	2370107	234	26559	10128	137079
**bin_60**	96.56	3.88	3582635	1052	7165	3405	42703
**bin_34**	96.55	2.23	4276665	475	29451	9003	115018
**bin_119**	96.48	3.85	2418564	481	48306	5028	184071
**bin_81**	96.45	1.48	3113672	393	47279	7922	207005
**bin_24**	96.32	2.50	4743587	657	21463	7220	84181
**bin_88**	96.31	1.42	2958917	655	7492	4517	37384
**bin_10**	96.29	4.03	5421633	1178	33462	4602	244428
**bin_51**	96.09	1.79	3793107	591	13879	6418	79388
**bin_110**	95.89	1.85	2554447	267	23331	9567	76948
**bin_28**	95.86	6.37	4513838	970	9642	4653	70580
**bin_76**	95.85	2.76	3246143	715	15294	4540	91324
**bin_59**	95.85	7.62	3587634	968	7179	3706	43956
**bin_36**	95.75	3.13	4242730	542	22472	7827	80523
**bin_4**	95.57	6.40	6886351	1668	13379	4128	134124
**bin_172**	95.56	1.24	1775061	197	41082	9010	128647
**bin_125**	95.50	3.12	2364686	564	7027	4192	26015
**bin_158**	95.25	3.58	1943329	208	25139	9342	85704
**bin_20**	94.83	3.09	4946263	655	25982	7551	101878
**bin_37**	94.81	0.38	4183242	791	17705	5288	89517
**bin_168**	94.76	1.71	1821186	189	28362	9635	146750
**bin_103**	94.71	2.14	2681028	478	10748	5608	32281
**bin_2**	94.44	7.41	7306097	1656	12937	4411	97239
**bin_163**	94.09	3.46	1920449	344	15479	5582	63387
**bin_131**	94.00	2.89	2318874	240	28803	9661	131715
**bin_127**	93.99	1.23	2343606	268	15126	8744	72655
**bin_130**	93.96	2.29	2322058	414	10463	5608	58565
**bin_156**	93.95	1.64	1953509	157	50189	12442	151913
**bin_182**	93.55	0.81	1679156	104	88735	16145	291316
**bin_45**	93.24	2.93	3858129	652	14193	5917	87250
**bin_162**	93.15	2.23	1928777	253	31279	7623	129799
**bin_23**	93.13	3.12	4821214	636	21233	7580	90026
**bin_160**	92.88	1.30	1938905	173	40336	11207	150108
**bin_170**	92.84	1.23	1799630	367	9462	4903	53645
**bin_126**	92.83	3.17	2355299	475	8231	4958	31074
**bin_75**	92.76	4.39	3297296	684	8631	4820	50138
**bin_120**	92.45	0.19	2409406	248	28541	9715	128628
**bin_71**	92.41	0.00	3369930	235	55757	14340	209217
**bin_133**	92.39	0.53	2301048	286	17298	8045	56500
**bin_35**	92.04	12.10	4263036	1812	4182	2352	33236
**bin_142**	91.83	1.34	2172109	217	46584	10009	129871
**bin_177**	91.39	4.79	1705246	510	5400	3343	29767
**bin_109**	90.69	6.46	2555319	663	6442	3854	26234

**bin_183**	90.49	2.55	1674721	351	9824	4771	40265
**bin_44**	89.86	2.24	3911892	449	42068	8712	225139
**bin_87**	89.68	5.97	2964270	1075	4328	2757	28974
**bin_143**	89.00	1.28	2154240	264	26516	8160	78797
**bin_53**	88.79	7.07	3765750	1308	5253	2879	31115
**bin_73**	88.44	3.11	3336298	803	10815	4154	120644
**bin_67**	87.92	5.05	3431998	1745	2626	1966	19806
**bin_46**	87.81	4.56	3837623	1018	6088	3769	58999
**bin_102**	87.63	5.05	2704238	1066	3863	2536	22986
**bin_154**	87.42	4.10	1981765	315	15593	6291	59577
**bin_52**	87.28	4.44	3790024	1105	5695	3429	58373
**bin_171**	87.23	1.64	1777890	198	18597	8979	88075
**bin_152**	87.17	10.26	2007809	430	10654	4669	33730
**bin_148**	87.02	5.88	2049690	922	3453	2223	24101
**bin_147**	86.73	15.22	2053475	915	3182	2244	34235
**bin_166**	86.49	1.28	1881285	265	14968	7099	62210
**bin_141**	86.44	0.73	2201638	209	29644	10534	89592
**bin_241**	85.71	0.00	923403	139	11418	6643	89918
**bin_83**	85.59	2.91	3061483	1444	3029	2120	40490
**bin_64**	85.51	6.48	3501084	1097	6405	3191	33599
**bin_128**	85.40	5.18	2340522	1043	3081	2244	18487
**bin_167**	84.90	4.99	1879418	739	3847	2543	22498
**bin_93**	84.29	9.14	2873275	1120	4169	2565	31919
**bin_47**	82.92	6.66	3824568	1645	3428	2324	28828
**bin_78**	82.86	6.12	3175624	1589	2783	1998	18163
**bin_138**	82.46	7.00	2262794	1088	2922	2079	22030
**bin_164**	80.92	4.47	1912093	654	4909	2923	18828
**bin_146**	77.45	1.59	2071338	846	3924	2448	23621
**bin_165**	77.02	0.70	1894867	293	21511	6467	72815
**bin_79**	75.99	6.23	3154721	1696	2595	1860	20737
**bin_40**	74.66	5.95	4120047	1318	5285	3125	41246
**bin_90**	73.79	4.78	2917076	1990	1801	1465	12759
**bin_161**	73.52	0.00	1934612	306	14612	6322	47276
**bin_65**	70.87	4.96	3481672	2602	1562	1338	53990
**bin_49**	70.82	8.07	3803033	2380	2083	1597	28447
**bin_82**	70.18	3.42	3100476	2124	1753	1459	14327
**bin_94**	69.01	5.76	2833797	1535	2429	1846	31942
**bin_108**	68.91	4.63	2565914	1211	3037	2118	29075
**bin_199**	67.39	7.28	1420450	333	8154	4265	26461
**bin_153**	62.07	0.00	1994836	172	55744	11597	198576
**bin_144**	61.91	3.85	2153320	1481	1796	1453	17242
**bin_184**	60.58	4.11	1649453	576	4276	2863	21411
**bin_137**	60.28	3.16	2277447	1651	1652	1379	12408
**bin_139**	59.84	2.91	2227482	470	8215	4739	50838
**bin_99**	57.27	0.00	2732739	1757	1948	1555	13539

**bin_186**	56.14	0.00	1638813	220	19186	7449	55138
**bin_192**	55.38	0.00	1571400	128	46685	12276	137919
**bin_201**	54.40	0.75	1410248	899	2030	1568	27953
**bin_118**	50.00	6.03	2433246	732	7126	3324	42446
**bin_179**	49.12	0.88	1695555	243	15217	6977	50228
**bin_169**	48.56	0.00	1816471	153	43573	11872	118017
**bin_178**	46.49	0.00	1698169	162	35423	10482	102699
**bin_215**	43.11	3.28	1209245	1204	1101	1004	5698
**bin_189**	42.81	3.02	1622096	1340	1392	1210	8009
**bin_194**	42.23	12.39	1554872	1613	987	963	6737
**bin_202**	41.69	0.94	1353370	89	35488	15206	133906
**bin_220**	41.54	12.07	1184172	1164	1077	1017	7549
**bin_232**	40.83	1.72	1006488	1048	1003	960	7698
**bin_205**	40.35	0.00	1301836	281	37395	4632	219725
**bin_247**	40.27	1.86	875460	394	3300	2221	17895
**bin_213**	39.89	3.09	1219287	1078	1256	1131	8001
**bin_198**	37.92	0.00	1428854	120	69433	11907	132928
**bin_228**	36.84	0.00	1039520	578	2143	1798	23926
**bin_207**	36.84	1.75	1285628	273	10733	4709	48908
**bin_204**	36.84	1.75	1346354	167	22228	8062	64291
**bin_196**	34.48	0.63	1548656	326	11704	4750	67528
**bin_48**	34.47	4.74	3805697	3635	1094	1046	12713
**bin_255**	33.78	1.72	772958	153	10235	5052	35919
**bin_214**	33.45	0.00	1219173	353	104123	3453	246251
**bin_254**	33.33	0.00	794385	50	42633	15887	90360
**bin_236**	32.26	0.00	945252	83	40023	11388	153862
**bin_231**	31.90	1.72	1023500	464	3034	2205	18868
**bin_129**	31.88	0.73	2330931	221	26952	10547	79566
**bin_212**	31.83	0.00	1239119	997	1403	1242	14483
**bin_191**	31.83	1.25	1602046	738	2852	2170	17600
**bin_218**	31.58	18.42	1193712	813	1617	1468	131842
**bin_136**	30.36	6.75	2286379	710	8787	3220	62127
**bin_216**	29.98	0.13	1205554	161	19686	7487	57806
**bin_237**	29.82	0.00	931156	161	13242	5783	61750
**bin_219**	29.82	0.00	1193318	226	17029	5280	47751
**bin_188**	29.75	0.63	1627878	176	37551	9249	170272
**bin_200**	29.31	0.00	1413067	374	6954	3778	40919
**bin_149**	29.31	0.00	2042882	382	10833	5347	41003
**bin_180**	28.07	1.75	1691813	1058	2034	1599	14973
**bin_249**	27.59	0.00	861684	227	6373	3795	30762
**bin_217**	26.32	0.00	1204637	161	18971	7482	85612
**bin_181**	25.95	0.46	1687247	160	29659	10545	113940
**bin_261**	25.44	1.75	704356	401	2250	1756	9623
**bin_243**	25.38	2.07	896508	587	1808	1527	27808

**bin_159**	25.37	0.00	1940013	214	40254	9065	102574
**bin_175**	25.32	0.10	1748849	219	16195	7985	108200
**bin_265**	24.12	2.59	613000	672	902	912	8017
**bin_225**	23.75	0.00	1067595	1154	975	925	5077
**bin_190**	22.80	1.35	1617060	347	13043	4660	79730
**bin_173**	22.59	8.79	1759545	1100	2036	1599	18086
**bin_245**	22.41	0.00	891933	128	17324	6968	93193
**bin_224**	21.95	0.00	1121195	1081	1042	1037	33548
**bin_260**	21.71	1.72	734485	740	1065	992	5735
**bin_230**	21.05	5.26	1024620	333	5193	3076	32926
**bin_187**	20.17	0.00	1631511	1222	1557	1335	11291
**bin_251**	20.01	1.51	831460	960	877	866	3849
**bin_229**	18.97	0.00	1032354	116	26856	8899	115088
**bin_195**	18.27	1.75	1553844	1626	992	955	12818
**bin_176**	17.53	0.16	1728689	874	2702	1977	16796
**bin_262**	16.85	0.00	703235	429	2348	1639	20769
**bin_209**	16.38	1.72	1273607	1099	1242	1158	40642
**bin_252**	15.79	1.75	818232	71	56848	11524	114763
**bin_234**	15.79	3.51	975248	297	5909	3283	37599
**bin_211**	15.52	0.00	1241251	345	7847	3597	47463
**bin_239**	15.22	0.00	929618	887	1113	1048	5531
**bin_248**	15.09	0.00	861882	190	10066	4536	51594
**bin_264**	14.04	3.51	615061	177	5111	3474	15348
**bin_244**	14.04	0.00	894003	391	3468	2286	27909
**bin_267**	13.79	1.72	588813	475	1438	1239	10541
**bin_238**	12.50	0.00	929992	218	130233	4266	210705
**bin_197**	12.50	0.00	1512476	170	20472	8896	100475
**bin_185**	12.50	0.00	1641026	337	13435	4869	80002
**bin_233**	12.08	0.00	991930	131	19944	7571	60488
**bin_1**	11.99	2.96	10941619	12217	852	895	20885
**bin_263**	11.93	0.00	696957	85	15284	8199	53129
**bin_68**	11.57	9.58	3423805	3277	1025	1044	33237
**bin_242**	11.44	0.00	905454	428	2912	2115	11811
**bin_250**	10.53	3.51	832899	245	5597	3399	39203
**bin_240**	10.37	2.24	929028	291	6798	3192	49383
**bin_210**	10.17	0.00	1259288	1168	1107	1078	14988
**bin_259**	9.87	0.02	739019	142	35505	5204	86564
**bin_208**	9.31	0.00	1282882	806	1977	1591	20230
**bin_222**	8.62	0.00	1161679	197	48952	5896	126278
**bin_253**	8.33	0.00	818056	310	3563	2638	22621
**bin_221**	8.33	0.00	1171751	318	5836	3684	32600
**bin_203**	8.33	0.00	1353011	288	10815	4697	43392
**bin_174**	8.33	0.00	1750914	345	43947	5075	144318
**bin_256**	8.01	0.53	748781	161	13391	4650	67693
**bin_266**	7.96	0.17	606917	178	5860	3409	43242

**bin_257**	6.13	0.51	745404	212	6275	3516	24177
**bin_235**	5.58	0.00	960563	848	1177	1132	17702
**bin_258**	5.52	0.00	742910	720	1052	1031	9670
**bin_270**	5.17	3.45	552539	174	6207	3175	41013
**bin_227**	5.17	1.72	1041478	1098	949	948	9034
**bin_86**	4.83	0.00	3028303	3255	937	930	8243
**bin_271**	4.17	0.00	512326	61	20689	8398	58478
**bin_193**	4.17	0.00	1566947	1495	1096	1048	8003
**bin_269**	0.00	0.00	560972	526	1129	1066	24776
**bin_268**	0.00	0.00	564750	69	19287	8184	55668
**bin_246**	0.00	0.00	884819	590	1815	1499	27238
**bin_226**	0.00	0.00	1061327	1093	995	971	9141
**bin_223**	0.00	0.00	1157384	310	83509	3733	169814
**bin_206**	0.00	0.00	1286057	362	6581	3552	61304
